# Does hyperthermic intraoperative chemotherapy lead to improved outcomes in patients with ovarian cancer? A single center cohort study in 111 consecutive patients

**DOI:** 10.1186/1754-9493-6-12

**Published:** 2012-06-15

**Authors:** Rene Warschkow, Ignazio Tarantino, Jochen Lange, Sascha A Müller, Bruno M Schmied, Michael Zünd, Thomas Steffen

**Affiliations:** 1 Department of Surgery, Kantonsspital St. Gallen (KSSG), St. Gallen, CH-9007, Switzerland; 2 Department of Surgery, Zuger Kantonsspital, Baar, CH-6340, Switzerland; 3 Institute of Medical Biometry and Informatics, University of Heidelberg, Heidelberg, D- 69120, Germany

**Keywords:** Cytoreductive surgery, Peritonectomy, Hyperthermic intraperitoneal chemotherapy, Epithelial ovarian cancer, Peritoneal carcinomatosis

## Abstract

**Background:**

For recurrent disease or primary therapy of advanced ovarian cancer, cytoreductive surgery (CRS) followed by adjuvant chemotherapy is a therapeutic option. The aim of this study was to evaluate the outcome for patients with epithelial ovarian cancer treated with hyperthermic intraoperative chemotherapy (HIPEC) and completeness of cytoreduction (CC).

**Methods:**

Data were retrospectively collected from 111 patients with recurrent or primary ovarian cancer operated with the contribution of visceral surgical oncologists between 1991 and 2006 in a tertiary referral hospital.

**Results:**

Ninety patients received CRS and 21 patients CRS plus HIPEC with cisplatin. Patients with complete cytoreduction (CC0) were more likely to receive HIPEC. Overall, 19 of 21 patients (90.5 %) with HIPEC and 33 of 90 patients (36.7 %) with CRS had a complete cytoreduction (P < 0.001). Incomplete cytoreduction was associated with worse survival rates with a hazard ratio (HR) of 4.4 (95%CI: 2.3-8.4) for CC1/2 and 6.0 (95%CI: 2.9-12.3) for CC3 (P < 0.001). In a Cox-regression limited to 52 patients with CC0 a systemic concomitant chemotherapy (HR 0.3, 95%CI: 0.1-0.96, P = 0.046) but not HIPEC (HR 0.98 with 95 % CI 0.32 to 2.97, P = 0.967) improved survival. Two patients (9.5 %) developed severe renal failure after HIPEC with absolute cisplatin dosages of 90 and 95 mg.

**Conclusions:**

Completeness of cytoreduction was proved to be crucial for long-term outcome. HIPEC procedures in ovarian cancer should be performed in clinical trials to compare CRS, HIPEC and systemic chemotherapy against CRS with systemic chemotherapy. Concerning the safety of HIPEC with cisplatin, the risk of persistent renal failure must be considered when dosage is based on body surface.

## Introduction

Epithelial ovarian cancer is primarily diagnosed in the advanced stage III or IV, as defined by FIGO (International Federation of Gynaecology and Obstetrics), and 75 % of these patients present with peritoneal carcinomatosis as a typical finding [1]. Epithelial ovarian cancer is second only to breast cancer as a leading cause of gynaecologic cancer-related mortality, with an estimated 14,500 deaths and an estimated 21,550 new cases in 2009 in the United States [2,3]. Disease-free survival rarely exceeds 18 months after standard therapy [4-6]. The recent 2009 statistics from the American Cancer Society report an overall survival of 46 %, compared to a rate of 37 % in 1975 [3].

For recurrent disease or primary therapy of advanced ovarian cancer (FIGO III and IV), cytoreductive surgery (CRS) followed by an adjuvant chemotherapy is the standardized therapeutic option [7]. CRS in combination with hyperthermic intraperitoneal chemotherapy (HIPEC) has also proved feasible, although it is associated with morbidity rates ranging from 0 % to 40 % and mortality rates ranging from 0 % to 10 % [8-11]. In patients with optimal cytoreduction and HIPEC, a 5-year survival rate ranging from 12 % to 66 % has been reported. Therefore, HIPEC after CRS was proposed as the “up-front treatment” for ovarian cancer with peritoneal carcinomatosis [1], which was substantially criticized by others [12]. The current state of knowledge mandates the use of extensive surgery whenever possible in primary and recurrent ovarian cancer [13,14]. Ibeanu *et al.* recommended that surgeons strive for "no residual disease” as often as possible to improve survival rates [9]. A residual tumour mass smaller than 2.5 mm is stipulated for the application of HIPEC [11]. To date, no phase III randomised prospective trial has been published confirming a benefit of the aggressive treatment strategy of CRS followed by HIPEC [8]. In addition, identifying which patients will undergo optimal cytoreduction and consequently qualify for HIPEC remains difficult [9]. Therefore, the abundant heterogeneity in study populations cannot be prevented because of individual patient selection for HIPEC by operating surgeons and the presumably variable and individually determined surgical aggressiveness applied to gain complete cytoreduction. Nevertheless, results of this treatment modality have been shown to be beneficial for patients with peritoneal carcinomatosis from appendix cancer, applying the same criteria for cytoreduction and methods for HIPEC [15-17].

The aim of this study was to assess the benefit from CRS with and without HIPEC and completeness of cytoreduction (CC) in a consecutive cohort of patients with advanced (FIGO III or IV) or recurrent ovarian cancer with involvement of visceral oncological surgeons for CRS. The primary endpoint of this study was the survival of patients. Secondary endpoints were comparison of CRS alone *vs.* CRS and HIPEC concerning morbidity and mortality.

## Patients and methods

### Patient selection

The present study is a retrospective single-institution observational study. A computer search of the institutional database was performed. Briefly, we identified 224 patients with ovarian cancer. Of these patients, 113 were excluded due to the lack of involvement of visceral oncological surgeons. The remaining 111 patients underwent interdisciplinary operations with the involvement of visceral oncological surgeons between 1991 and 2006. These patients had histologically confirmed primary advanced ovarian cancer (FIGO III or FIGO IV) or recurrent ovarian cancer (independent of initial FIGO stage).

### Data collection

Data pertaining to patients’ demographics, operative details, postoperative mortality, morbidity and histological results were gathered retrospectively from medical files. Mortality was defined as any death occurring during the 30 days following surgery. Follow-up data were obtained from medical records and by contacting the general practitioners by telephone.

### Selection criteria for HIPEC

General inclusion criteria for HIPEC were histologically confirmed primary (FIGO IIIc or IV) or recurrent ovarian carcinoma (independent of initial FIGO stage), age between 18 and 70 years, ASA stage II or III, informed consent including explicit consent about the experimental nature of the HIPEC procedure, adequate renal function with a renal function of >50 ml/min, no serious medical condition, no hypersensitivity to cisplatin and no symptomatic peripheral neuropathy. In patients with primary advanced ovarian cancers FIGO IIIc that were initially inoperable due to their poor general condition, neoadjuvant chemotherapy with three cycles of taxol or carboplatin was applied. If the patient’s condition improved (clinical impression), HIPEC was performed. Patients with primary ovarian carcinoma FIGO IV did not generally qualify for HIPEC except with fully regressive malignant pleural effusion under chemotherapy with taxol or carboplatin. Patients with recurrent disease were included in the absence of a distant metastasis. In patients with primary or recurrent disease where CC0 or CC1 was not reached intraoperatively, cytoreduction without HIPEC was performed. The treatment modality for each individual patient was determined preoperatively by an interdisciplinary tumor board with participation from the following departments: gynaecology, visceral surgical oncology, medical oncology, radiology, radio oncology, and pathology.

### Dosage of cisplatin in HIPEC

In general, there was a body surface related cisplatin dose of 50 mg/m^2^ in HIPEC. The body surface area was calculated according to Mosteller [18]. In patients with preoperative renal insufficiency or previous carboplatin treatment, the dosage was reduced by 30 %.

### Treatment modality

Peritonectomy was performed as proposed by Sugarbaker [11]. CC was assessed according to Sugarbaker: CC0: no residual disease; CC1: residual disease with nodules measuring less than 2.5 mm; CC2: residual disease with nodules measuring between 2.5 mm and 2.5 cm; and CC3: residual nodules greater than 2.5 cm [11]. All patients received perioperative antibiotic prophylaxis and anticoagulation routinely to prevent deep vein thrombosis with low molecular weight heparin.

In patients with HIPEC, CRS was followed by immediate direct instillation of heated platinum-based chemotherapy to address residual tumour masses [19]. A RanD Performer^TM^ LRT device [RanD S.r.l., Medolla (MO), Italy] was used. During HIPEC, an intra-abdominal temperature of 42 °C was targeted, and the duration of the HIPEC was 90 minutes after the target temperature was reached. After reaching the target temperature, a surgeon mixed the intraabdominal fluid constantly to maintain the target temperature. The temperatures were recorded every 10 min in the HIPEC protocol. In all patients, renal function was monitored during the HIPEC procedure. If renal function decreased, patients were hydrated and furosemide was given.

### Statistical analysis

Statistical analysis was performed using SPSS 11.5 software. Continuous data are expressed as mean ± standard deviation (range). A two-sided *P* value < 0.05 was considered to indicate statistical significance. Confidence intervals (95 % CI) of binominal proportions were estimated according to a Wilson method [20]. Survival time was calculated from the time of operation. For univariate survival analysis, log-rank tests were performed. For multivariate survival analysis, a full Cox-regression model with additional variable selection was applied. The R statistical software using the bootStepAIC package was used for bootstrapping the backward variable selection process from the full Cox-regression model [21].

### Authorization

The analysis of patient data was approved by the Swiss Federal Expert Commission for Physician Confidentiality. Further, this study was approved by the local ethical commission.

## Results

### Patient selection

From 1991 to 2006, a total of 111 patients with either advanced (FIGO ≥ III) or recurrent ovarian cancer were treated with an interdisciplinary approach at a tertiary referral hospital with the contribution of visceral surgical oncologists and gynaecologists. A total of 21 (19 %) of the 111 patients had CRS and HIPEC and 90 (81 %) had CRS only. Table 1 lists the baseline characteristics of the two groups. No statistically significant differences in these characteristics were identified.

**Table 1:** Baseline characteristics

		**Total**	**HIPEC; N = 21**	**Operation only; N = 90**	***P***
Disease	Primary disease	53 (47.7%)	10 (47.6%)	43 (47.8%)	0.990 ^B)^
	Recurrent disease**	58 (52.3%)	11 (52.4%)	47 (52.2%)	
Recurrent disease: time since diagnosis	Months	40.9 ± 44.2	51.7 ± 70	38.3 ± 36.3	0.960 ^A)^
Initial/actual FIGO stage^C)^	FIGO IIa	1 (0.9%)	1 (4.8%)	0 (0%)	0.638 ^A)^
	FIGO IIc	4 (3.6%)	2 (9.5%)	2 (2.2%)	
	FIGO IIIa	1 (0.9%)	0 (0.0%)	1 (1.1%)	
	FIGO IIIb	11 (9.9%)	2 (9.5%)	9 (10.0%)	
	FIGO IIIc	72 (64.9%)	11 (52.4%)	61 (67.8%)	
	FIGO IV	22 (19.8%)	5 (23.8%)	17 (18.9%)	
Age at operation	Years	63.8 ± 12.1	58.9 ± 11.4	65.0 ± 12.0	0.064 ^A)^
Hospitalization time	Days	28.5 ± 14.3	32.9 ± 18.2	27.5 ± 13.1	0.292 ^A)^
Body mass index	kg/m²	25.3 ± 5.9	25.7 ± 4.7	25.1 ± 6.2	0.441 ^A)^
Follow-up, survivors	Months	35.6 ± 35.3	29.4 ± 15.4	38.3 ± 40.9	0.824 ^A)^
ASA stage ^D)^	I	1 (0.9%)	0 (0.0%)	1 (1.1%)	0.103 ^A)^
	II	71 (64.0%)	17 (81.0%)	54 (60.0%)	
	III	38 (34.2%)	4 (19.0%)	34 (37.8%)	
	IV	1 (0.9%)	0 (0.0%)	1 (1.1%)	
Initial grading	G1	7 (6.3%)	3 (14.3%)	4 (4.4%)	0.414 ^A)^
	G2	30 (27.0%)	5 (23.8%)	25 (27.8%)	
	G3	65 (58.6%)	13 (61.9%)	52 (57.8%)	
	G4	3 (2.7%)	0 (0.0%)	3 (3.3%)	
	GX	1 (0.9%)	0 (0.0%)	1 (1.1%)	
Initial tumor growth pattern	**Serous**	87 (78.4%)	16 (76.2%)	71 (78.9%)	0.484 ^B)^
	Mucinous	2 (1.8%)	1 (4.8%)	1 (1.1%)	
	Endometrioid	10 (9.0%)	3 (14.3%)	7 (7.8%)	
	Clear cell	2 (1.8%)	0 (0.0%)	2 (2.2%)	
	Other	4 (3.6%)	0 (0.0%)	4 (4.4%)	
Concomitant radiation		4 (3.6%)	0 (0.0%)	4 (4.4%)	0.325 ^B)^
Concomitant chemotherapy	Total	75 (67.6%)	14 (66.7%)	61 (67.8%)	0.922 ^B)^
	Carboplatin	56 (50.5%)	13 (61.9%)	43 (47.8%)	0.244 ^B)^
	Taxol	14 (12.6%)	4 (19.0%)	10 (11.1%)	0.324 ^B)^
	Endoxan	4 (3.6%)	0 (0.0%)	4 (4.4%)	0.325 ^B)^
	Other	20 (18.0%)	2 (9.5%)	18 (20.0%)	0.216 ^B)^

Mean ± standard deviation

N (%)^A)^ Mann–Whitney *U*-Test

^B)^ Chi-square test

^C)^ initial FIGO stage for recurrences and actual FIGO stage for primary disease

FIGO: International Federation of Gynecology and Obstetrics

^D)^ ASA: American Society of Anesthesiologists

### Operative and peri-operative outcome

The mean operation time was significantly longer in the HIPEC group at 8.2 ± 1.6 h (range: 4.3–11 h) compared to 4.5 ± 1.7 h in the group without HIPEC (range: 1.5–9.0 h) (*P* < 0.001). While all patients in the HIPEC group were postoperatively admitted to the intensive care unit, significantly fewer patients of the non-HIPEC group were admitted to the intensive care unit [21/21 (100 %) *vs.* 65/90 (72.2 %); *P* = 0.007]. The mean number of resected peritoneum areas was significantly higher in the HIPEC group at 4.5 ± 1.8 (range: 1–7) compared to 2.0 ± 1.0 (range: 0–7) in the group without HIPEC (*P* < 0.001). Additionally, the mean number of organs involved in the resection was significantly higher in the HIPEC group at 5.5 ± 1.7 (range: 2–8) compared to 1.7 ± 4.0 (range: 1–9) in the group without HIPEC (*P* < 0.001). For the more extensive resections, significantly better CC scores were observed in patients in the HIPEC group: 19 patients (90.5 %) had a CC0 score, 2 patients (9.5 %) had a CC1 or CC2 score, and none of the patients who had HIPEC had a CC3 score. In the group without HIPEC, 33 patients (36.7 %) had a CC0 score, 37 patients (41.1 %) a CC1 or CC2 score, and 20 patients (22.7 %) had a CC3 score (*P* < 0.001). The mean body surface related dosage of cisplatin in HIPEC was 40.2 ± 7.8 mg/m^2^ with a range of 29.6-50.9 mg/m^2^. In 11 out of 21 patients receiving HIPEC, a body surface related cisplatin dose of about 50 mg/m^2^ was generally administered. In the other 10 patients with HIPEC, the cisplatin dose was reduced by 30 % due to preoperative renal insufficiency (3 patients), previous carboplatin treatment (4 patients) or both (3 patients). The mean absolute dosage of cisplatin was 69.8 ± 14.6 mg with a range of 50–95 mg.

### Morbidity and mortality

In the HIPEC group, 6/21 patients (28.6 %, 95 % CI: 13.6 %–50.2 %) developed surgical complications compared to 24/90 patients (26.7 %, 95 % CI: 18.6 %–36.7 %) in the group without HIPEC (*P* = 0.784). In the HIPEC group, 7/21 patients (33.3 %, 95 % CI: 17.0 %–54.8 %) developed general complications compared to 33/90 (36.7 %, 95 % CI: 27.4 %–47.0 %) in the group without HIPEC (*P* = 0.985). Ten patients in the group without HIPEC died within 30 days after their operation (11.1 %, 95 % CI: 6.0 %–19.4 %), while no patients in the HIPEC group died within the first 30 days after their operation (95 % CI: 0.0 %–18.2 %) (*P* = 0.131). The causes of death were cardial or pulmonary insufficiency in four patients, sepsis in three patients, multiorgan failure in two patients, and a pulmonary embolism in one patient. In the HIPEC group, 3/21 patients (14.3 %, 95 % CI: 4.1 %–35.5 %) required a second operation compared to 13/90 patients (14.4 %, 95 % CI: 8.5 %–23.3 %) in the group without HIPEC (*P* = 0.985). Major surgical complications in the HIPEC group were postoperative bleeding (N = 1), wound infection (N = 4), and anastomotic leakage (N = 1). In addition to pulmonary complications (N = 4), two patients in the HIPEC group (9.5 %) developed severe persistent renal failure. Further analysis revealed a significant correlation with the absolute dosage of cisplatin but not with the body surface related dosage. Mean absolute dosage of cisplatin in the two patients with persistent renal failure was 92.5 ± 3.5 mg (range: 90–95 mg) compared to 67.4 ± 13.2 mg (range: 50–90 mg) (*P* = 0.026). The body surface related dosage of cisplatin in the two patients with persistent renal failure was 46.7 ± 2.1 mg (range: 45.2–48.2 mg) compared to 39.5 ± 7.8 mg (range: 29.6–50.9 mg) (*P* = 0.338).

### Survival analysis

**Figure 1:**
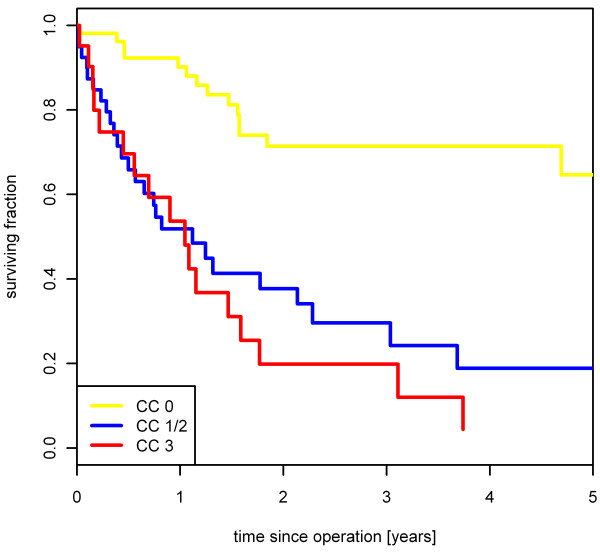
**Influence of completeness of cytoreduction on survival.** Completeness of cytoreduction correlates significantly with survival (N = 111; *P* < 0.001).

**Figure 2:**
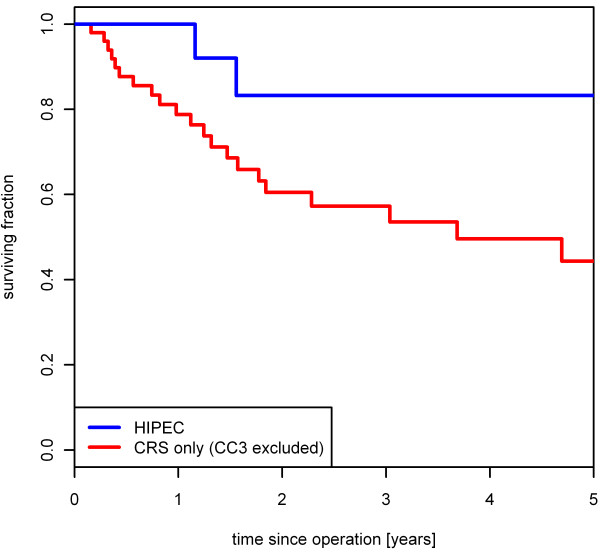
**Patient benefit from HIPEC.** In the HIPEC group, 2- and 5-year survival rates were each 72.5%, compared to patients with CRS only (CC3 score excluded) with 2- and 5-year survival rates at 52.0% and 38.3%, respectively (N = 70; *P* = 0.043).

For the 52 patients with a CC0 score, the 2- and 5-year survival rates were 71.0 % and 63.0 %, respectively. For the 39 patients with a CC1 or CC2 score, the 2- and 5-year survival rates were 36.7 % and 17.0 %, respectively. For the remaining 20 patients with a CC3 score, the 2-year survival rate was 17.5 % whereas no patient survived 5 years (*P* < 0.001) (Figure 1). Incomplete cytoreduction was associated with significantly worse survival rates in Cox regression analysis with a hazard ratio (HR) of 4.4 (95%CI: 2.3-8.4) for CC1/2 and 6.0 (95%CI: 2.9-12.3) for CC3. No relevant difference in the 5-year survival rate was found concerning the patients undergoing a primary operation (N = 53) *versus* those with recurrent disease (N = 58) (*P* = 0.944, 5-year survival of 36.0 % *versus* 32.7 %). In the HIPEC group, the 2- and 5-year survival rates were each 72.5 %. This outcome was compared to that for patients with CRS only after excluding patients with a CC3 score. The 2- and 5-year survival rates in this subgroup were 52.0 % and 38.3 %, respectively (N = 70; *P* = 0.043). (Figure 2)

To evaluate the influence of the cisplatin dosage on survival, patients with HIPEC were post hoc divided into high- and low-dose groups dichotomised at the median absolute dosage of 70 mg. There was no significant difference in 5-year survival rates between these two groups (70.0 % *versus* 76.2 %; *P* = 0.691).

Finally, a multivariate Cox-regression analysis was performed. This analysis was limited to the 52 patients with CC0 score (19 with HIPEC and 33 without HIPEC) to avoid a bias from the collinearity of HIPEC and CC score. In this analysis, systemic chemotherapy was the only significant predictor for survival (P = 0.038). HIPEC (*P* = 0.830), primary operation *versus* operation after relapse (*P* = 0.349), initial FIGO stage (*P* = 0.964), and age (*P* = 0.541) did not significantly influence survival. In a limited Cox-regression model, HIPEC (HR 0.98 with 95 % CI 0.32 to 2.97, P = 0.967) did not add a predictive value for survival to systemic chemotherapy (HR: 0.33 with 95 % CI 0.12 to 0.96, P = 0.046). A lack of significant predictive value of HIPEC for survival was confirmed in a stepwise backward and forward variable selection procedure. In contrast, a bootstrapping procedure of the backward variable selection indicated a beneficial effect of HIPEC for survival. HIPEC was selected in 89.19 % of 9999 permutated samples as a relevant predictor with a beneficial effect in 99.97 % (Table 2).

**Table 2:** Multivariate Cox-regression model and bootstrapping

	**Cox regression model ^A)^**				**Bootstrap ^B)^**	
	*P*^C)^	HR	95.0% CI for HR		Selection (%)	HR > 1 (%)
Age [years]	0.541	1.016	0.966	1.069	94.75	100.00
Systemic chemotherapy	0.038	0.279	0.084	0.932	90.32	0.00
Recurrent disease	0.349	0.558	0.163	1.908	30.83	15.21
FIGO IV *vs.* III/II	0.964	0.964	0.199	4.676	21.60	63.29
HIPEC	0.830	0.881	0.275	2.820	89.19	0.03

Analysis limited to 52 patients with CC0 score

^A)^ Full Cox-regression model. Additionally, backward and forward variable selections identified systemic chemotherapy as the only independent significant predictor for survival

^B)^ Bootstrapping procedure for backward variable selection process with 9999 samples. Column selection expresses the fraction indicating how often the variable was selected in the backward variable selection process and column HR > 1 expresses the fraction of a hazard ratio above 1.0

^C)^ Likelihood ratio test

## Discussion

This study confirmed CC, systemic concomitant chemotherapy, and age as significant independent factors influencing the survival rate. According to previously published literature, incomplete cytoreduction (CC scores > CC0) was associated with the worst outcome reflected by a HR of 4.7 in patients with CC1 or CC2 and a HR of 5.3 in patients with CC3, respectively [13,22,23]. This finding suggests that all efforts must target maximal cytoreduction. As already shown in a meta-analysis [14], the benefit of maximal cytoreduction in the present study was not limited to primary disease; the same effect was found in patients with recurrent disease. While HIPEC appeared to significantly improve survival rates in univariate analysis, no independent benefit of HIPEC on survival was demonstrated on multivariate analysis when limiting it to the 52 patients with CC0 score to exclude a bias from collinearity between CC score and HIPEC. This lack of evidence for a benefit of HIPEC on survival is in contrast to previous findings from various studies reporting also “non-randomised experience” [12]. However, repeating the statistical variable selection process in 9999 permutated samples of the original data, HIPEC was selected in 89 % as a predictor for survival and was associated with a beneficial effect in more than 99 %. This finding suggests that the lacking influence of HIPEC in conventional analysis might be caused by low power and collinearity [21].

The presented data inherits a strong selection bias in favour to HIPEC. Complete cytoreduction was significantly more often achieved in patients receiving HIPEC. As a tendency, patients with HIPEC were younger and presented with less advanced ASA stages. Mortality was limited to patients not receiving HIPEC. This selection bias may well explain the significant benefit of HIPEC on survival in univariate analysis. On the other hand, the lack of significance in multivariate analysis might theoretically be attributed to a small sample size. In addition, it is important to note that this study was limited to a single centre cohort that underwent operations between 1991 and 2006 at a “low volume” hospital for HIPEC in ovarian cancer. As the present study has a retrospective observational study design, manifold other forms of bias cannot be ruled out, although partly anticipated in multivariate analyses. Nevertheless, there is some evidence that results from observational studies are often similar to those of clinical trials [24]. Also, in the light of the recently developed Comparative Effectiveness Research (CER), observational studies are valuable as they might help clinicians in their decision process.

Morbidity was comparable in patients with and without HIPEC. Operation time was significantly longer in the HIPEC group, even taking into account about 2 h for performing a HIPEC procedure. Moreover, patients with HIPEC were more likely to be admitted to the intensive care unit. Therefore, the potential benefit of HIPEC has to be weighted critically against the additional effort.

The safety analysis revealed two patients with renal failure after HIPEC with a high absolute cisplatin dosage of 90 mg and 95 mg, respectively. It is important to consider the relevant risk of renal failure after HIPEC and to avoid overdosage, especially as there was no dose-dependent survival benefit in this study [25]. Although there is a correlation between body surface and blood volume, the pharmacodynamics of drugs dosed by the body surface is still highly variable and thus dosing on the body surface is increasingly considered controversial for systemic administration [26]. For HIPEC, dosing by the body surface is even more questionable, given that the aim is the highest possible drug concentration in the peritoneum without undue local and systemic toxicity [27]. Since the amount of cytotoxic drug is fixed by dosing on the body surface, the effective concentration in the perfusate can vary considerably between patients [28,29]. Pharmacokinetic analyses have shown that reducing the concentration of the cytotoxic drug in the perfusate reduces the efficacy even if the amount of the drug remains the same [30]. Concerning the safety of cisplatin dosing, our data suggest a harmful effect, even at the body surface related dose of 50 mg/m² cisplatin. Due to the occurrence of renal failure after administration of the usual cisplatin dose a prospective study, “HIPEC of Recurrent Ovarian Cancer - A Feasibility Study” (NCT00968799), was initiated and is presently recruiting.

## Conclusion

In conclusion, this study has provided some evidence indicating that the completeness of cytoreduction is crucial for a long-term positive outcome. The removal of all evident macroscopic disease should be the goal of primary and secondary cytoreductive surgery in ovarian cancer. The present study failed to identify an independent benefit of HIPEC. Until evidence-based prove of significant therapeutic benefit, HIPEC procedures in ovarian cancer should be embedded in clinical trials favourably comparing CRS, HIPEC and systemic chemotherapy against CRS with systemic chemotherapy only. Finally, for HIPEC, the dosing regime should be carefully evaluated.

## Competing interests

The authors declare that they have no competing interests.

## Authors’ contributions

TS, RW, BMS and MZ conceived the study design. TS and RW drafted the manuscript. RW and IT performed the statistical analysis. MZ supervised the data acquisition. MZ, SAM, BMS and JL critically revised the manuscript for important intellectual content. All authors read and approved the final manuscript.
